# Optimizing nourishment: nutritional knowledge, practices, and cultural beliefs among postpartum lactating mothers in Saudi Arabia: a cross-sectional study

**DOI:** 10.3389/fpubh.2026.1804312

**Published:** 2026-04-14

**Authors:** Asmaa Mohamed Ali AlAbd, Salwa Ali Marzouk, Aziza Ibrahim Mohamed, Mahmoud Abdelwahab Khedr, Abdulhafith Alharbi, Nahed Mousa Saber, Sara Farhan Alenizi, Amany Ali Abd Elsalam

**Affiliations:** 1 Psychiatric and Mental Health Nursing, College of Nursing, University of Hail, Hail, Saudi Arabia; 2 Maternal and Child Health, College of Nursing, University of Hail, Hail, Saudi Arabia; 3 Nursing Department, College of Applied Medical Science, University of Bisha, Bisha, Saudi Arabia; 4 College of Nursing, University of Hafr Al-Batin, Hafar Al-Batin, Saudi Arabia

**Keywords:** postpartum mothers, lactation, nutritional knowledge, dietary practices, cultural nutrition beliefs

## Abstract

**Background:**

Adequate maternal nutrition in the postpartum period is crucial for maternal recovery, optimal breast milk composition, and healthy infant development. Nutritional knowledge and culturally influenced dietary practices play a key role in shaping mothers’ dietary behaviors. This study aimed to assess nutritional knowledge, practices, and cultural food beliefs among postpartum lactating mothers in Saudi Arabia.

**Methods:**

A cross-sectional study was conducted among 442 postpartum mothers within 6 months after delivery, recruited using a two-stage cluster random sampling technique from maternal and child health centers in Bisha and Ha’il cities. A standardized interviewer-administered questionnaire encompassing sociodemographic traits, nutritional knowledge, dietary habits, and culturally influenced eating habits was used to gather data. To find determinants of adequate dietary practices and strong nutritional awareness, binary logistic regression analysis was used.

**Results:**

Overall, 54.8% of mothers were classified as having good nutritional practices, and 50.9% were classified as having good nutritional knowledge. Good nutritional knowledge was significantly associated with older maternal age, higher educational attainment, and employment status. Good nutritional practices were significantly associated with employment, better income status, and planned pregnancy. Regarding cultural beliefs, 65.8% of mothers consumed culturally prepared traditional foods, 63.6% avoided certain foods based on cultural beliefs, and 69.2% participated in celebratory food practices following childbirth. Herbal tea, puerperium nuts, and Moghate were the most commonly consumed traditional foods.

**Conclusion:**

Although more than half of postpartum mothers reported good dietary practices, only half demonstrated adequate nutritional knowledge. To improve maternal and infant nutritional outcomes in Saudi Arabia, culturally sensitive, evidence-based nutritional education programs must be integrated into prenatal and postnatal healthcare services.

## 1 Introduction

The postpartum period, often referred to as the “fourth trimester,” is a critical stage for both the mother and the newborn, characterized by substantial physical, emotional, and nutritional demands. Adequate nutrition during lactation is essential for maternal recovery, immune function, and the production of high-quality breast milk, thereby supporting the health and well-being of both mother and infant (1, 2). This period also represents an important window of opportunity for nutritional intervention, as maternal dietary habits during lactation may have lasting implications for maternal health and infant development (3).

Lactating mothers require increased energy intake and specific nutrients to support milk production and maintain their own health (4). Macronutrients and micronutrients, including proteins, fats, vitamins, and minerals, are all important for maternal nutritional status and breast milk quality (5). In Saudi Arabia, maternal and child health is a national priority under Saudi Vision 2030, providing strong justification for integrating nutrition-focused strategies into routine postpartum care (6). In addition, the Saudi Ministry of Health has emphasized maternal and child health promotion, including awareness of appropriate nutrition during lactation (7).

Nutrition knowledge is a key component of healthy dietary behavior, although knowledge alone may not be sufficient to ensure sustained dietary change. According to Social Cognitive Theory, health behavior is shaped not only by knowledge, but also by self-efficacy, social support, and environmental influences (8). A systematic review has shown that awareness of dietary guidelines is positively associated with healthier eating habits and improved diet quality, while limited health literacy is associated with poorer health outcomes (9). Therefore, understanding the relationship between nutritional knowledge and actual dietary behavior is essential for developing effective evidence-based strategies in maternal and public health nutrition (10).

Cultural beliefs are another important determinant of food behavior, shaping not only what individuals eat, but also how, when, and why certain foods are consumed. These beliefs are often deeply rooted in social norms, traditions, and family practices (11). Traditional food beliefs and dietary practices during pregnancy, childbirth, and the postpartum period differ across cultures and may strongly influence maternal nutrition-related decisions (12).

In Saudi Arabia, postpartum women commonly follow culturally specific dietary practices and beliefs related to certain foods, beverages, and lifestyle behaviors after childbirth (13). For example, foods such as dates are often consumed because of their perceived nutritional and lactation-promoting benefits (14). Herbal remedies and food restrictions are also commonly followed based on family traditions and cultural expectations (15). However, some traditional postpartum dietary practices may not fully align with evidence-based nutritional recommendations and may, in some cases, affect maternal dietary adequacy and breast milk quality (16).

The postpartum period in Saudi Arabia is culturally significant and is often marked by specific dietary customs intended to promote maternal recovery and lactation, such as the consumption of warm soups, dates, and energy-rich foods, alongside avoidance of foods perceived as “cold” or harmful (17). Despite rapid modernization and healthcare reforms in line with Saudi Vision 2030, these traditional beliefs continue to influence maternal food choices. Understanding how such cultural beliefs interact with nutritional knowledge and dietary practices is therefore important for designing effective and culturally sensitive maternal nutrition interventions.

To the best of our knowledge, limited research in Saudi Arabia has comprehensively examined postpartum mothers’ nutritional knowledge, dietary practices, and culturally rooted food beliefs within the same analytical framework, particularly across different regions. This represents an important gap in maternal nutrition research. Therefore, the present study aimed to investigate nutritional knowledge and practices among postpartum lactating mothers in Bisha and Hail cities in Saudi Arabia and to assess the cultural beliefs influencing food preferences and food avoidances. The findings may provide useful evidence for improving postpartum nutrition support within this unique cultural context.

## 2 Materials and methods

### 2.1 Study design and setting

A descriptive cross-sectional study was conducted at maternal and child health (MCH) clinics in the Saudi Arabian cities of Hail and Bisha over 7 months, from mid-June 2024 to mid-January 2025. These clinics provide obstetric and maternal health services to a large proportion of women during the perinatal period.

### 2.2 Study population

The study targeted postpartum mothers. Eligible participants were women aged 18–45 years, within 6 months postpartum, and actively breastfeeding or expressing milk for their infants. Mothers were excluded if they had medical conditions that could interfere with breastfeeding, such as severe chronic illness or infection, or if their infants had congenital anomalies or serious health conditions. Eligibility was assessed before recruitment using self-report and available medical information from the MCH facilities. Mothers were invited to participate only if they met all inclusion criteria and none of the exclusion criteria.

### 2.3 Sample size and sampling technique

The sample size was calculated using OpenEpi software (18). The calculation was based on a 95% confidence level, 5% absolute precision, and an expected prevalence of nutritional knowledge of 50% to maximize the sample size. After accounting for a 15% non-response rate, the final sample size was set at 442 mothers. Participants were recruited using a two-stage cluster random sampling technique to ensure representation across the two study regions. First, four MCH centers were randomly selected, two from Bisha and two from Hail. Second, within each selected center, a systematic random sample of 10 mothers per day was selected on randomly chosen data collection days using the attendance list as the sampling frame.

### 2.4 Study tools

Data were collected using a pre-validated structured questionnaire administered through face-to-face interviews with the selected mothers. The questionnaire included the following domains:

1 Sociodemographic and perinatal characteristics

This section included age, educational level, occupational status, income, region of residence, parity, history of abortion, regular antenatal follow-up during the most recent pregnancy, and whether the most recent pregnancy was planned or unplanned.

2 Nutritional knowledge

Nutritional knowledge was assessed using a structured tool consisting of 14 statements related to postpartum nutrition. Responses were scored as “No” = 1 and “Yes” = 2 for each item. The total score ranged from 14 to 28. Participants scoring <20 were classified as having poor knowledge, whereas those scoring ≥20 were classified as having good knowledge. These cut-off values were used as operational thresholds to facilitate the interpretation of participants’ overall nutritional knowledge and to distinguish relatively good from poor levels of knowledge.

3 Nutritional practices

Nutritional practices were assessed using a tool focusing on mothers’ actual dietary behaviors and consisting of 9 statements. Responses were scored as Practice Always (PA) = 2, Practice Rarely (PR) = 1, and Do Not Practice (DP) = 0. Mothers who reported consuming the specified food items daily were classified as Practice Always, whereas those consuming them only 1–2 times per week were classified as Practice Rarely. Based on the total score, nutritional practice was categorized as good practice (9–18) or poor practice (0–8). These cut-off values were used as operational thresholds to facilitate the interpretation of postpartum dietary practices and to distinguish good from poor levels of practices among the participants.

4 Cultural food beliefs and behaviors

This section assessed cultural beliefs and behaviors influencing postpartum food choices, including traditional food preferences and restrictions, use of herbal remedies, perceptions of foods considered beneficial or harmful after childbirth, and the consumption of celebratory foods during the postpartum period.

### 2.5 Validity and reliability

The questionnaire used to assess knowledge and practice was originally developed by Kayoed et al. (19) to evaluate maternal nutrition during pregnancy. For the present study, the items were modified to reflect the postpartum period. Content validity of the adapted questionnaire was assessed by five experts in public health and maternal and child health, and revisions were made based on their feedback before data collection. Reliability was evaluated using Cronbach’s alpha, which yielded coefficients of 0.91 for the knowledge section and 0.76 for the practice section.

### 2.6 Pilot study

After development of the tool, a pilot study was conducted on 22 postpartum women from two MCH centers in the Hail and Bisha regions to assess the clarity, relevance, and comprehensibility of the study tools and to estimate the time required for completion. These participants were excluded from the final analysis.

### 2.7 Ethical considerations

This study received ethical approval from the Institutional Review Board of the College of Nursing, University of Hail (H-2024-383, dated March 6, 2024). Administrative permission was also obtained from the managers of the participating MCH centers. All procedures were conducted in accordance with the ethical principles of the Declaration of Helsinki and its subsequent revisions. Participants were informed that participation was voluntary, that they had the right to ask questions and withdraw at any stage without consequences, and that all collected data would be kept confidential and used solely for research purposes. Oral informed consent was obtained before participation.

### 2.8 Data collection procedures

An official letter was obtained from the College of Nursing, University of Hail, and submitted to the managers of the selected MCH centers to request permission for data collection. Coordination meetings were conducted with nurse managers to organize the sampling process.

The researcher attended the selected MCH centers on the designated data collection days from 10:00 a.m. to 2:00 p.m. On each day, eligible mothers were identified from the daily attendance lists using a systematic random sampling approach. During the interview, the researcher introduced herself and briefly explained the purpose and nature of the study, then obtained oral informed consent. Each participant was interviewed individually in Arabic for approximately 15–20 min, and responses were recorded using the study questionnaire. On average, 30 women were interviewed per week, and data collection was completed over 4 months.

### 2.9 Data management and statistical analysis

Data were analyzed using IBM SPSS Statistics for Windows, Version 22.0 (20). Normality of quantitative variables was assessed using the Kolmogorov–Smirnov test. Quantitative variables were summarized using mean ± standard deviation, while qualitative variables were presented as frequencies and percentages. Associations between categorical variables were examined using the chi-square test or the Monte Carlo exact test when more than 20% of expected cell counts were less than 5. Binary logistic regression analysis was performed to identify predictors of nutritional knowledge and practices. Adjusted odds ratios (AORs) with 95% confidence intervals (CIs) were reported. Statistical significance was set at *p* ≤ 0.05.

## 3 Results

### 3.1 Sociodemographic and perinatal characteristics

A total of 442 postpartum lactating mothers participated in this study. The study population was primarily composed of Saudi nationals within the 30–40-year age bracket. Most participants were homemakers with a university-level education and reported an adequate household income. Regarding perinatal history, the majority of mothers were multiparous, and most had attended antenatal follow-ups during their most recent pregnancy. However, a significant proportion reported that their pregnancy had not been planned, as detailed in Table 1.

**Table 1:** Sociodemographic and perinatal characteristics of postpartum lactating mothers (*N* = 442).

Variable	Category	*n*	%
Age group (years)	20–30	73	16.6
	30–40	276	62.4
	>40	93	21.0
Study setting	Hail	287	64.9
	Bisha	155	35.1
Educational level	Secondary	59	13.3
	University	234	52.9
	Postgraduate	149	33.8
Occupation	Housewife	315	71.3
	Employed	127	28.7
Household income	Inadequate	51	11.6
	Adequate	382	86.4
	Adequate and safe	9	2.0
Nationality	Saudi	408	92.3
	Non-Saudi	34	7.7
Parity	1–2 births	24	5.4
	>2 births	418	94.6
History of abortion	No	309	69.9
	Yes	133	30.1
Antenatal care	No	72	16.3
	Yes	370	83.7
Pregnancy planning	No	282	63.8
	Yes	160	36.2

Data are presented as numbers (*n*) and percentages (%).

### 3.2 Postpartum nutritional knowledge and practices

**Figure 1:**
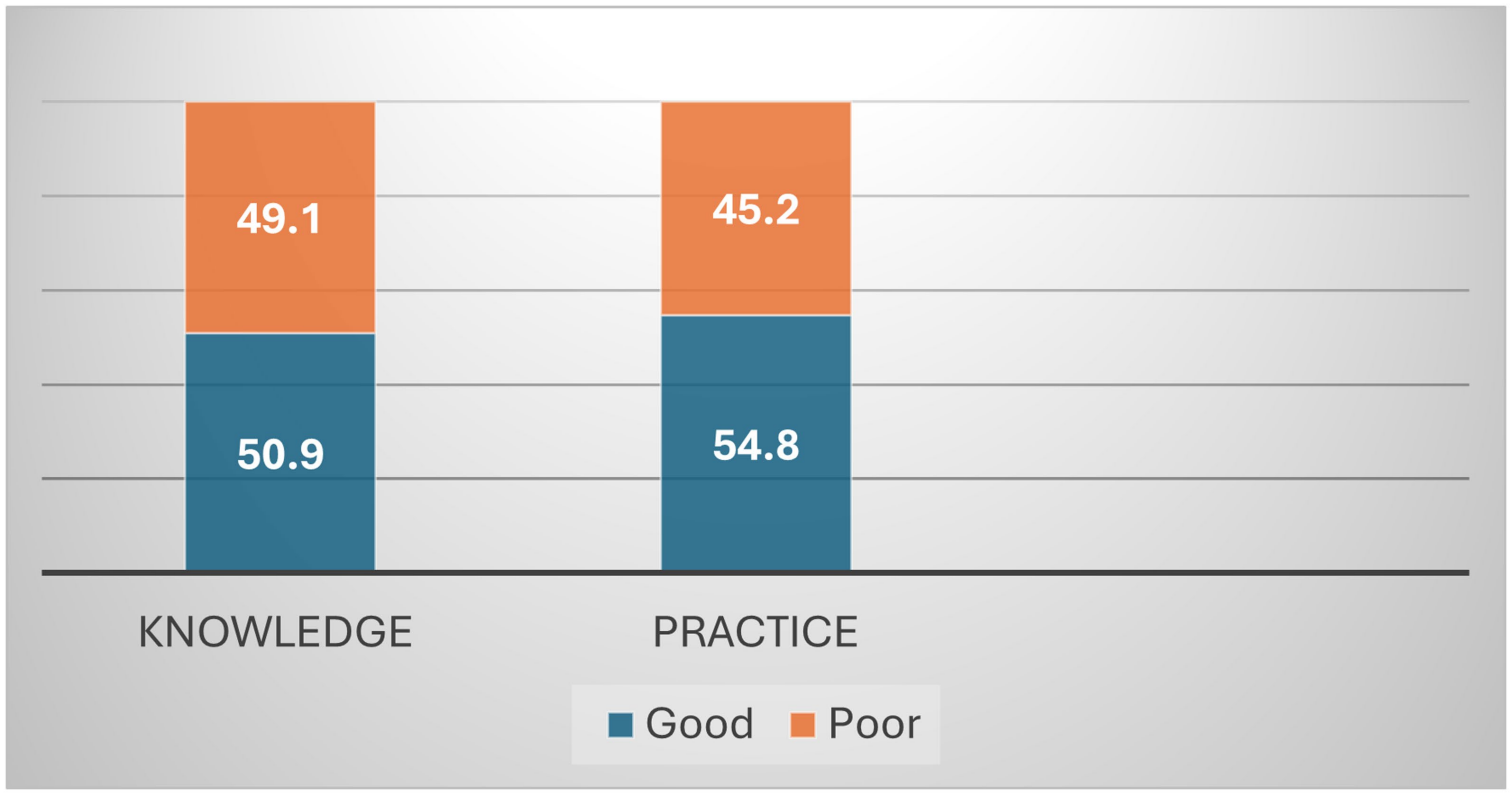
Classification of postpartum nutritional knowledge and practices.

Table 2 and Figure 1 present the levels of nutritional knowledge and practices among lactating mothers. Approximately half of the participants were classified as having good nutritional knowledge. The mean knowledge score was 20.08 ± 2.59, which fell within the predefined “good knowledge” category based on the study cut-off (≥20). However, the distribution shown in Figure 1 indicates a nearly balanced classification between good and poor nutritional knowledge categories. Slightly more than half of the participants were classified as having good nutritional practices. The mean practice score was 9.07 ± 2.47.

**Table 2:** Postpartum nutritional knowledge and practice scores among lactating mothers (*N* = 442).

Variable	Mean ± SD	Minimum–Maximum
Nutritional knowledge score	20.08 ± 2.59	16–27
Nutritional practice score	9.07 ± 2.47	3–16

SD: standard deviation.

### 3.3 Cultural beliefs and traditional food practices

**Figure 2:**
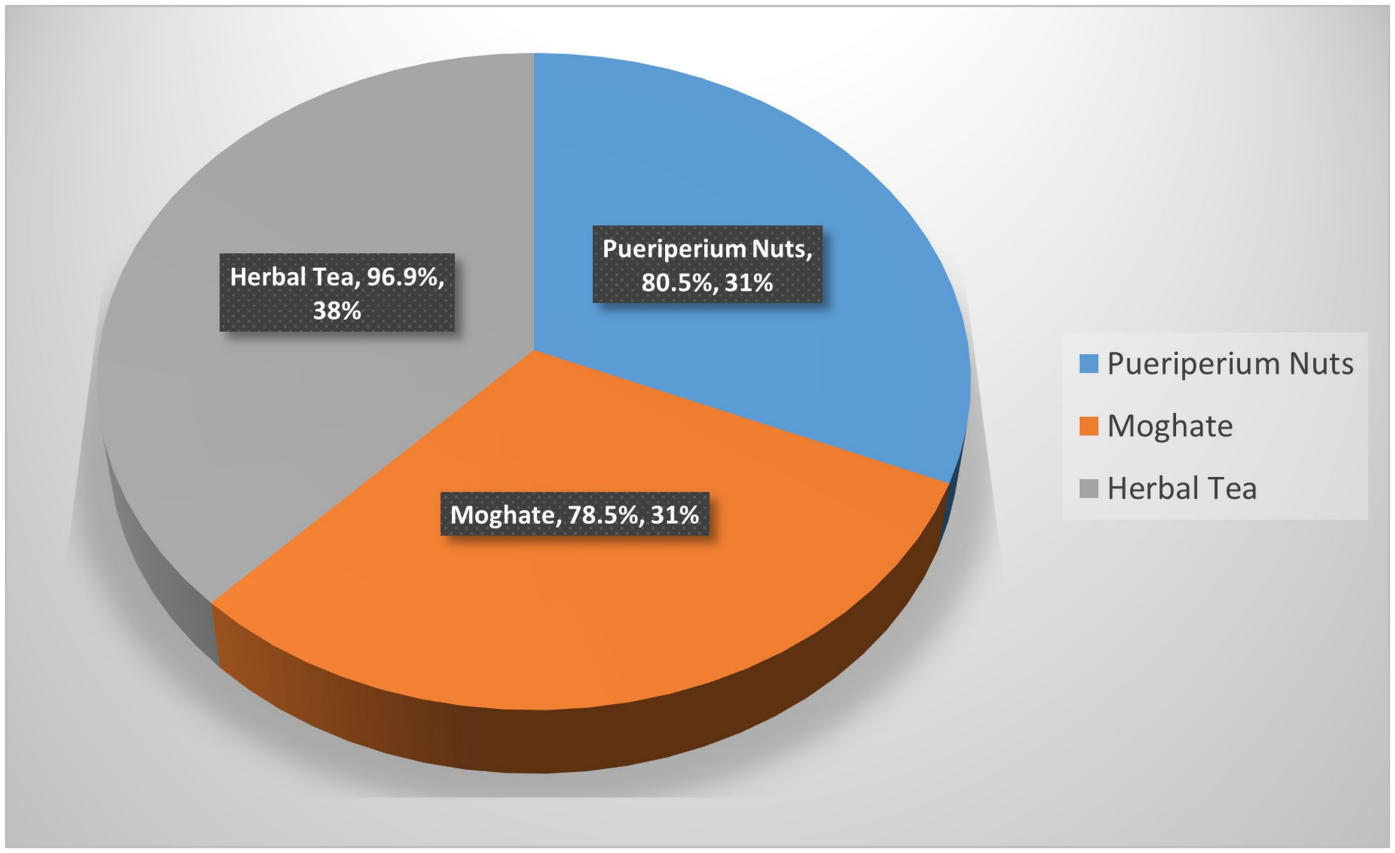
Traditional celebratory foods consumed during the postpartum period.

Table 3 shows the cultural beliefs and traditional food practices reported by the studied mothers. A majority of mothers reported consuming culturally prepared traditional foods and participating in culturally celebratory food practices following childbirth. Conversely, food avoidance based on cultural beliefs was also prevalent. As illustrated in Figure 2, herbal tea was the most commonly consumed traditional item, followed by puerperium nuts and Moghate.

**Table 3:** Cultural beliefs and traditional food practices among postpartum lactating mothers (*N* = 442).

Item	Yes, *n* (%)	No, *n* (%)
Consumption of culturally prepared traditional foods	291 (65.8)	151 (34.2)
Avoidance of certain foods due to cultural beliefs	281 (63.6)	161 (36.4)
Consumption of celebratory foods after childbirth	306 (69.2)	136 (30.8)

Data are presented as numbers (*n*) and percentages (%).

### 3.4 Postpartum food preferences and avoidances

**Figure 3:**
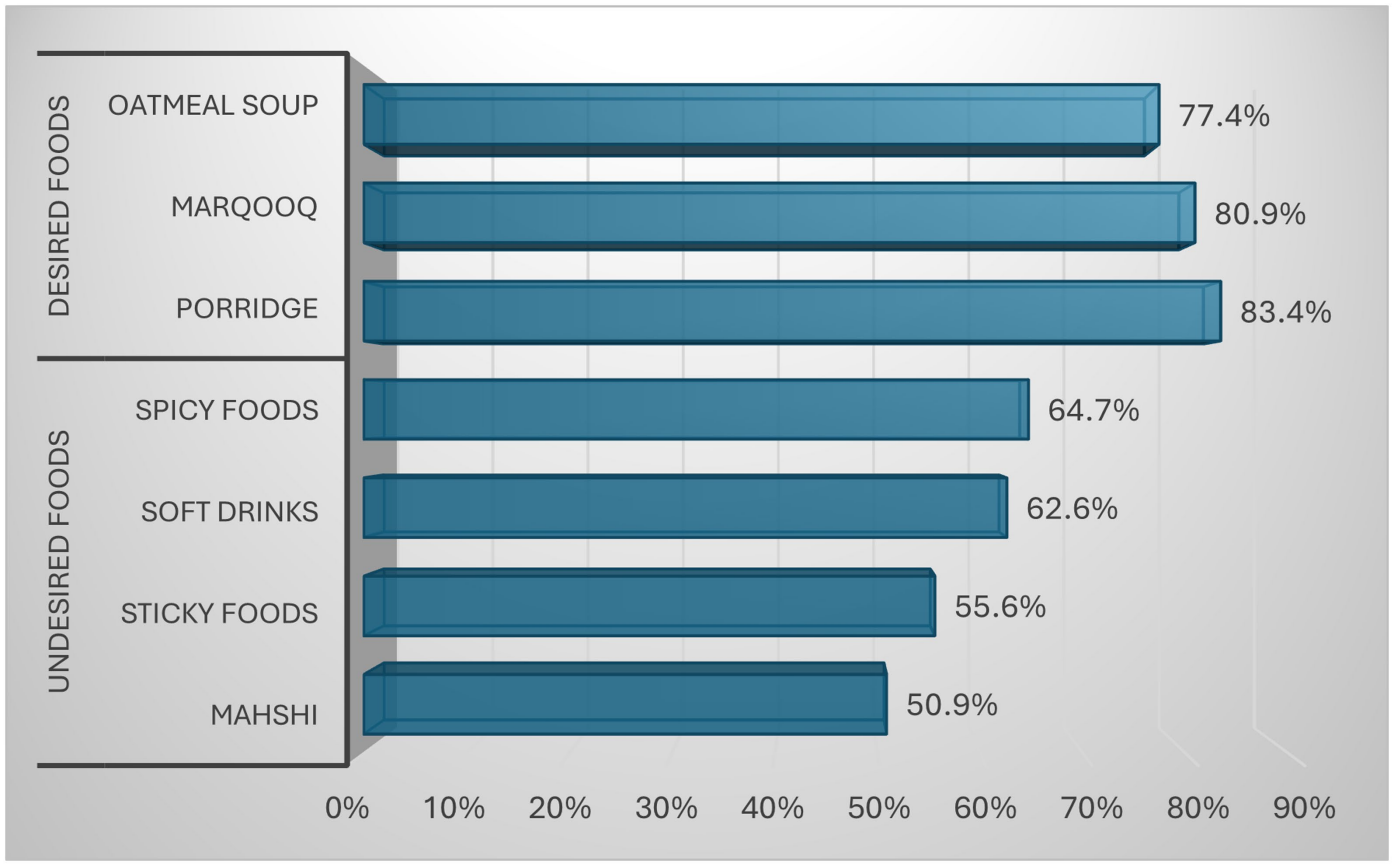
Postpartum food preferences and avoidances among lactating mothers.

Figure 3 presents postpartum food preferences and avoidances among lactating mothers. Preferred traditional items included porridge, Marqouq, and oatmeal soup. In contrast, many mothers reported avoiding spicy foods and soft drinks, while approximately half of the mothers avoided sticky foods and Mahshi. These findings highlight the coexistence of culturally preferred foods and food avoidance practices during the postpartum period.

### 3.5 Predictors of postpartum nutritional knowledge

Table 4 shows the predictors of good postpartum nutritional knowledge among the studied mothers. Age was significantly associated with nutritional knowledge, as mothers aged 30–40 years were more likely to have good nutritional knowledge than those aged 20–30 years (AOR = 2.98). Regarding educational level, mothers with university or postgraduate degrees demonstrated significantly higher odds of good nutritional knowledge compared to those with secondary education. However, the exceptionally high adjusted odds ratios and wide confidence intervals for these categories are identified as statistical artifacts. This inflation results from the very low frequency of good nutritional knowledge within the reference group (secondary education), rather than a lack of sample robustness in the higher education groups. Additionally, employment status and the MCH setting were also identified as significant predictors of maternal nutritional knowledge, while other demographic variables did not show statistically significant associations.

**Table 4:** Binary regression analysis of predictors of good postpartum nutritional knowledge among lactating mothers.

Variables	Category	Knowledge		Sig.	AOR	95% C. I. for AOR	
		Poor	Good				
		No (%)	No (%)			Lower	Upper
Age in years	20–30	56 (76.7)	17 (23.3)		Ref		
	30–40	121 (43.8)	155 (56.2)	0.003	2.98	1.43	6.18
	>40	40 (43.0)	53 (57.0)	0.071	2.28	0.93	5.59
MCH setting	Hail	130 (45.3)	157 (54.7)		Ref		
	Bisha	87 (56.1)	68 (43.9)	0.006	0.28	0.11	0.69
Educational level	Secondary	58 (98.3)	1 (1.7)		Ref		
	University	114 (48.7)	120 (51.3)	0.000	49.88^a^	5.70^a^	436.24^a^
	Postgraduate	45 (30.2)	104 (69.8)	0.000	322.73^a^	35.46^a^	2937.73^a^
Occupation	Housewife	202 (64.1)	113 (35.9)		Ref		
	Employed	15 (11.8)	112 (88.2)	0.000	16.57	7.77	35.34
Income	Inadequate	28 (54.9)	23 (45.1)		Ref		
	Adequate	187 (49.0)	195 (51.0)	0.778	1.13	0.47	2.74
	Adequate and safe	2 (22.2)	7 (77.8)	0.176	4.04	0.53	30.49
Nationality	Saudi	199 (48.8)	209 (51.2)		Ref		
	Non-Saudi	18 (52.9)	16 (47.1)	0.545	0.75	0.29	1.91
Parity	1–2	12 (50.0)	12 (50.0)		Ref		
	>2	205 (49.0)	213 (51.0)	0.614	1.32	0.45	3.85
Abortion	No	157 (50.8)	152 (49.2)		Ref		
	Yes	60 (45.1)	73 (54.9)	0.395	1.29	0.71	2.34
Antenatal follow-up	No	34 (47.2)	38 (52.8)		Ref		
	Yes	183 (49.5)	187 (50.5)	0.909	1.05	0.47	2.33
Pregnancy planning	No	144 (51.1)	138 (48.9)		Ref		
	Yes	73 (45.6)	87 (54.4)	0.955	1.02	0.59	1.75

Statistical significance was set at *p* ≤ 0.05. AOR, adjusted odds ratio; CI, confidence interval; Ref., reference category.

^a^The inflated Adjusted Odds Ratios (AOR) values for university and postgraduate levels are a statistical artifact caused by the very low frequency of good knowledge in the reference category (secondary education, *n* = 1), despite the robust sample size in the higher education groups. These estimates indicate a very strong association but should be interpreted with caution as point estimates.

### 3.6 Predictors of postpartum nutritional practices

Table 5 shows the predictors of postpartum nutritional practices among the studied mothers. In terms of nutritional practices, both income and employment status emerged as significant predictors. Mothers in the highest income category showed substantially higher odds of good practices compared to those with inadequate income; however, this specific estimate should be interpreted with caution due to the limited number of participants in that subgroup. Employment status also positively influenced practice levels. In contrast, factors such as age, educational level, and parity did not significantly predict postpartum nutritional practices in this study.

**Table 5:** Binary regression analysis of predictors of good postpartum nutritional practices among lactating mothers.

Variables	Category	Practice		Sig.	AOR	95% C. I. for AOR	
		Poor	Good				
		No (%)	No (%)			Lower	Upper
Age in years	20–30	34 (46.6)	39 (53.4)		Ref		
	30–40	118 (42.8)	158 (57.2)	0.324	1.34	0.75	2.40
	>40	48 (51.6)	45 (48.4)	0.511	1.28	0.61	2.68
MCH setting	Hail	132 (46.0)	155 (54.0)		Ref		
	Bisha	68 (43.9)	87 (56.1)	0.707	0.88	0.46	1.69
Educational level	Secondary	32 (54.2)	27 (45.8)		Ref		
	University	106 (45.3)	128 (54.7)	0.797	0.91	0.42	1.94
	Postgraduate	62 (41.6)	87 (58.4)	0.758	1.116	0.55	2.25
Occupation	Housewife	164 (52.1)	151 (47.9)		Ref		
	Employed	36 (28.3)	91 (71.7)	0.000	2.51	1.52	4.13
Income	Inadequate	42 (82.4)	9 (17.6)		Ref		
	Adequate	157 (41.1)	225 (58.9)	0.000	6.97	2.98	16.29
	Adequate and safe	1 (11.1)	8 (88.9)	0.002	36.56^a^	3.59^a^	372.59^a^
Nationality	Saudi	184 (45.1)	224 (54.9)		Ref		
	Non-Saudi	16 (47.1)	18 (52.9)	0.730	0.87	0.40	1.89
Parity	1–2	10 (41.7)	14 (58.3)		Ref		
	>2	190 (45.5)	228 (54.5)	0.772	1.15	0.44	3.01
Abortion	No	143 (46.3)	166 (53.7)		Ref		
	Yes	57 (42.9)	76 (57.1)	0.736	0.92	0.57	1.49
Antenatal follow-up	No	39 (54.2)	33 (45.8)		Ref		
	Yes	161 (43.5)	209 (56.5)	0.427	1.32	0.67	2.60
Pregnancy planning	No	158 (56.0)	124 (44.0)		Ref		
	Yes	42 (26.3)	118 (73.8)	0.000	3.70	2.32	5.91

Statistical significance was set at *p* ≤ 0.05. AOR, adjusted odds ratio; CI, confidence interval; Ref., reference category.

^a^The high adjusted Odds Ratios (AOR) and wide confidence interval for the Adequate and safe income category are attributed to the small number of participants in this subgroup (*n* = 9). Therefore, this estimate should be interpreted with caution.

## 4 Discussion

A healthy diet during the postpartum period is essential for the long-term health and well-being of both mother and infant. Nurses play a central role in improving postpartum mothers’ nutritional knowledge through education, individualized counseling, and support for healthy dietary practices. This study examined nutritional knowledge and practices among postpartum mothers in Saudi Arabia during lactation. In addition to describing knowledge and practices, the study used multivariable logistic regression analysis to identify factors associated with better nutritional knowledge and dietary practices, thereby adding analytical depth to the findings.

The present study showed that nearly half of the mothers demonstrated good nutritional knowledge, while slightly more than half reported good nutritional practices. These findings indicate a generally acceptable, though not uniformly high, level of postpartum nutritional knowledge and practice among the studied mothers. Although the mean overall knowledge score fell within the predefined “good knowledge” category, responses across individual items showed variability, indicating that knowledge was stronger in some areas than in others. Therefore, the findings were interpreted as reflecting generally adequate overall knowledge, with remaining gaps in specific aspects of postpartum nutrition rather than uniformly good knowledge across all items. The slightly better practice level compared with knowledge may also suggest that some mothers follow beneficial dietary behaviors through family influence, routine advice, or cultural habit, even when detailed nutritional understanding is incomplete. Similar findings have been reported in previous studies. In Saudi Arabia, Alhashem et al. (17) identified suboptimal levels of postpartum nutritional knowledge among mothers in Riyadh, while Tessema et al. (21) found that lactating mothers showed 52.0% adequate knowledge compared with only 28.7% appropriate practices, highlighting the complexity of the relationship between knowledge and behavior.

Several maternal characteristics were significantly associated with nutritional knowledge. Mothers aged 30–40 years were more likely to demonstrate good nutritional knowledge than younger mothers. This finding is consistent with the assumption that older women may accumulate more health-related experience through repeated exposure to pregnancy, childbirth, infant feeding, and healthcare services. Comparable findings were reported by Bibi et al. (22), who found that older mothers tend to have more nutritional knowledge, as well as by Debela et al. (23), Papežová et al. (24), and Glick et al. (25). The consistency across diverse settings suggests that increased maternal age tends to coincide with higher nutrition literacy, possibly due to accumulated experience and greater engagement with healthcare systems.

Educational level was also significantly associated with nutritional knowledge. Mothers with university or postgraduate education had greater odds of good nutritional knowledge than those with only secondary education. This may be explained by the role of education in improving access to health information, comprehension of nutritional messages, and the ability to make informed nutrition-related decisions. Employment status was similarly associated with good knowledge. Working mothers may have broader social exposure, greater access to structured health information, and stronger opportunities to engage with health education messages. These findings are in line with Glick et al. (25), who found that nutrition-disease knowledge was higher among highly educated individuals. Likewise, Sanlier et al. (26) reported a substantial relationship between nutritional literacy and both advanced education and employment, while Marhamah et al. (27) revealed a positive association between employment status and nutritional knowledge in relation to nutritional status.

In contrast, no statistically significant association was found between postpartum nutritional knowledge and reproductive characteristics such as parity. This differs from the findings of Esmat et al. (28), who reported a weak but significant negative correlation between the number of children and maternal nutritional knowledge score. This discrepancy may be related to contextual differences in the study population, cultural setting, or measurement methods.

Regarding postpartum nutritional practices, employed mothers and those with better income status were significantly more likely to report good nutritional practices. This may reflect better financial capacity to obtain diverse and nutritious foods, as well as greater autonomy in food-related decisions. These findings are consistent with Teferi et al. (29), who found that employed mothers were less likely to have inadequate dietary diversity practices than housewives. In the present study, women with planned pregnancies were also more likely to report good nutritional practices. This may indicate that pregnancy planning is associated with greater readiness for maternal responsibilities, increased motivation to engage in healthy behaviors, and better use of antenatal and postnatal care services. This interpretation is supported by the Health Belief Model and the Theory of Planned Behavior (30, 31). However, because the present study used a cross-sectional design, these findings should be interpreted as associations rather than causal relationships. In addition, the relatively large adjusted odds ratio observed for mothers with adequate and safe income should be interpreted cautiously, as this category included a small number of participants, which may have reduced estimate stability and contributed to the wide confidence interval.

One of the major strengths of the present study is its focus on cultural food beliefs during the postpartum period. Most mothers reported engaging in culturally rooted food practices, including consumption of traditional foods, avoidance of certain foods because of prevailing beliefs, and participation in celebratory food rituals after childbirth. These findings suggest that traditional cultural practices remain highly influential in shaping postpartum dietary behavior and may, in some cases, be prioritized over formal nutritional guidance during the early postpartum period. This tendency appears to be associated with prevailing social norms that encourage adherence to elders’ recommendations, as well as beliefs that certain foods may cause infant discomfort. This interpretation aligns with the “hot and cold” concept described by Alhashem et al. (17).

Similarly, Olajide et al. (32) reported that nutrition advice sought from family members, friends, relatives, healthcare providers, and media sources influences food beliefs and practices during pregnancy and postpartum. While some women, particularly those of older age, may continue to adhere strongly to traditional beliefs, younger and more educated women may be more selective in following such practices. This reflects the dynamic interaction between cultural continuity and exposure to formal health information. The present study also showed that mothers preferred specific traditional postpartum foods such as porridge, Marqouq, and oatmeal soup. At the same time, many mothers reported avoiding spicy foods, soft drinks, and sticky foods such as Mahshi. These findings are important because traditional postpartum practices should not be viewed as uniformly harmful or uniformly beneficial. Some culturally preferred foods may support postpartum recovery by providing warmth, energy, hydration, or selected nutrients. However, certain restrictive practices may unintentionally reduce dietary diversity if they limit intake of important food groups without appropriate nutritional alternatives. Therefore, distinguishing between culturally supportive practices and potentially restrictive ones is essential for developing practical and respectful postpartum nutrition guidance.

Previous Saudi studies support these findings. Alhashem et al. (17) in Riyadh found that more than half of mothers increased their intake of traditional foods during the postpartum period, including black seed, Marqouq, and dates with Rashad, while women from the middle province favored oatmeal and fewer consumed Earika. Similarly, Lamadah (33) reported in Makkah that many women during the puerperium consumed specific herbs such as Almajelb and Anise, believing that they support lochial drainage, and consumed foods such as al farika to compensate for blood loss. These practices demonstrate that postpartum dietary choices are often embedded within culturally transmitted beliefs about healing and maternal recovery.

Comparable traditions have also been reported in other cultures. Ramulondi et al. (34) found that Zulu women in South Africa commonly consume soft maize-meal porridge during postpartum recovery for its perceived restorative value. Li et al. (35) documented that postpartum dietary practices in China and Korea emphasize porridge and nutrient-rich soups. In India, traditional preparations containing edible gum, dry nuts, and wheat are used to strengthen the back and reproductive organs, while herbal teas are consumed to support physical recuperation (36). In addition to preferred foods, avoidant practices are also reported across cultures. Jeong et al. (37) noted that spicy foods are avoided in Korea due to concerns about infant colic or digestive disturbances, while Tobing et al. (38) found that cold foods and soft drinks are restricted postpartum in Indonesia because they are believed to disturb internal balance and delay recovery. Taken together, these findings suggest that postpartum food behavior is strongly shaped by culturally transmitted beliefs about healing, body balance, lactation, and infant well-being.

Overall, the findings of this study suggest that postpartum nutrition education should be culturally sensitive and practically oriented. Healthcare providers should recognize that many mothers do not make food choices solely on the basis of biomedical advice, but also within a cultural and family context. Accordingly, postpartum nutrition counseling should aim to reinforce beneficial traditions while respectfully addressing practices that may limit dietary adequacy or diversity. Such an approach is more likely to be accepted by mothers and families and may improve the practical value of nutritional guidance during the postpartum period.

### 4.1 Strengths and limitations

This study has several strengths. First, data were collected through face-to-face interviews rather than self-administered online questionnaires, which likely improved response completeness and reduced misunderstanding of questionnaire items. Second, including mothers from Hail and Bisha provided contextual diversity and offered insight into postpartum nutritional knowledge, practices, and cultural food beliefs across different Saudi settings. Third, the use of multivariable logistic regression enhanced the analytical value of the study by identifying factors associated with postpartum nutritional knowledge and practices.

However, several limitations should be acknowledged. Because the study employed a cross-sectional design, temporal relationships could not be established, and causal inferences cannot be made. Therefore, the identified relationships should be interpreted as associations only. In addition, the study was conducted in selected cities, which may limit the generalizability of the findings to postpartum mothers in other regions of Saudi Arabia or in different sociocultural settings. The interviewer-administered approach, although useful for clarifying questions, may also have introduced response bias, including social desirability bias. Furthermore, some regression estimates were relatively high and should be interpreted with caution, particularly where certain comparison categories included small numbers of participants, which may have affected estimate stability and widened the confidence intervals. Finally, the lack of longitudinal follow-up limited the ability to assess changes in postpartum nutritional knowledge and dietary practices over time.

## 5 Conclusion

This study provides important insight into postpartum nutritional knowledge, practices, and food-related cultural beliefs among mothers in Hail and Bisha, Saudi Arabia. Approximately half of the mothers demonstrated good nutritional knowledge, and slightly more than half reported good nutritional practices. Older age, higher educational level, and employment were significantly associated with better nutritional knowledge, while employment, adequate income, and planned pregnancy were significantly associated with better nutritional practices. The study also highlighted the important role of cultural beliefs in shaping postpartum food preferences, food avoidance, and traditional dietary rituals.

These findings underscore the need for culturally sensitive postpartum nutrition education that respects women’s traditions while promoting dietary adequacy and evidence-based maternal care. Rather than disregarding cultural beliefs, healthcare providers should adopt a culturally competent and negotiation-based approach that supports beneficial practices and respectfully addresses those that may limit dietary diversity or nutritional balance. Special attention should be directed toward mothers with lower educational attainment and limited financial resources, as these groups may be more vulnerable to knowledge gaps or less favorable dietary practices. Because this study was cross-sectional, the findings should be interpreted as associations rather than causal relationships. Future longitudinal research is recommended to examine changes in postpartum nutritional knowledge and practices over time and to further explore how culturally shaped dietary behaviors may influence maternal and infant health outcomes.

## Data Availability

The raw data supporting the conclusions of this article will be made available by the authors, without undue reservation.

## References

[1] Victora CG Bahl R Barros AJD França GVA Horton S Krasevec J . Breastfeeding in the 21st century: epidemiology, mechanisms, and lifelong effect. Lancet. (2016) 387:475–90. doi: 10.1016/S0140-6736(15)01024-7, 26869575

[2] Carretero-Krug A Montero-Bravo A Morais-Moreno C Puga AM Samaniego-Vaesken ML Partearroyo T . Nutritional status of breastfeeding mothers and impact of diet and dietary supplementation: a narrative review. Nutrients. (2024) 16:301. doi: 10.3390/nu16020301 38276540 PMC10818638

[3] Bernier E Simoneau C Desroches S Morisset AS Robitaille J. Implementation of postpartum nutritional interventions in healthcare, community and eHealth: a systematic review. Matern Child Health J. (2024) 28:1897–910. doi: 10.1007/s10995-024-03985-5 39292385

[4] Marshall NE Abrams B Barbour LA Catalano P Christian P Friedman JE . The importance of nutrition in pregnancy and lactation: lifelong consequences. Am J Obstet Gynecol. (2022) 226:607–32. doi: 10.1016/j.ajog.2021.12.035, 34968458 PMC9182711

[5] Allen LH Donohue JA Dror DK. Limitations of the evidence base used to set recommended nutrient intakes for infants and lactating women. Adv Nutr. (2018) 9:295S–312S. doi: 10.1093/advances/nmy019, 29846528 PMC6008957

[6] Alarifi AM Alshahrani NZ Jokhdar H Asiri AM. Advancing health through sustainable development goals–Saudi Arabia’s mid-journey progress and insights. J Epidemiol Glob Health. (2025) 15:48. doi: 10.1007/s44197-025-00385-y, 40126702 PMC11933552

[7] Saudi Arabian health ministry (2024). Kingdom of Saudi Arabia - Ministry of Health Portal, Available online at: https://www.moh.gov.sa/en (Accessed August 22, 2024).

[8] Bandura A. Social Foundations of Thought and action: A social Cognitive Theory. Englewood Cliffs (NJ): Prentice-Hall (1986).

[9] Spronk I Kullen C Burdon C O'Connor H. Relationship between nutrition knowledge and dietary intake. Br J Nutr. (2014) 111:1713–26. doi: 10.1017/S0007114514000087 24621991

[10] Black RE Victora CG Walker SP Bhutta ZA Christian P De Onis M . Maternal and child undernutrition and overweight in low-income and middle-income countries. Lancet. (2013) 382:427–51. doi: 10.1016/S0140-6736(13)60937-X, 23746772

[11] Jayasinghe S Byrne NM Hills AP. Cultural influences on dietary choices. Prog Cardiovasc Dis. (2025) 90:22–26. doi: 10.1016/j.pcad.2025.02.003, 39921186

[12] Dubey A Chatterjee K Chauhan VS Sharma R Dangi A Adhvaryu A. Risk factors of postpartum depression. Ind Psychiatry J. (2021) 30:S127–31. doi: 10.4103/0972-6748.328803, 34908678 PMC8611548

[13] Kumar D Goel NK Pandey AK . Role of ayurveda and traditional medicine in postpartum care: a global perspective. J Tradit Complement Med. (2018) 8:369–74.

[14] Al-Shahib W Marshall RJ. The fruit of the date palm: its possible use as the best food for the future? Int J Food Sci Nutr. (2003) 54:247–59. doi: 10.1080/09637480120091982, 12850886

[15] Withers M Kharazmi N Lim E. Traditional beliefs and practices in pregnancy, childbirth and postpartum: a review of the evidence from Asian countries. Midwifery. (2018) 56:158–70. doi: 10.1016/j.midw.2017.10.019, 29132060

[16] Zareen H Mousa IA Okud A Malik MM Alsultan T Alnasser A . Dietary and behavioral practices in the postpartum period among Saudi women in the Kingdom of Saudi Arabia. Med Sci. (2023) 27:1–9. doi: 10.54905/disssi/v27i131/e42ms2616

[17] Alhashem AM Alrasheed FA Alwallan LK Almutairi MF Bin Khathran YM Alenzi YA . Traditional nutritional beliefs and practices among mothers in Riyadh during the puerperal period: a cross-sectional study. Int J Women's Health. (2025) 17:913–22. doi: 10.2147/IJWH.S484271, 40165858 PMC11955736

[18] Dean AG Sullivan KM Soe MM. OpenEpi: Open Source Epidemiologic Statistics for Public Health. (2013) Available online at: https://www.openepi.com/SampleSize/SSCohort.htm (Accessed February 12, 2024).

[19] Kayode OO Alabi QK Oshineye AO. Nutritional knowledge and practices among expectant mothers in Olorunda local government area, Osogbo, Osun state. Int J Food Sci Biotechnol. (2021) 6:66. doi: 10.11648/j.ijfsb.20210603.11

[20] IBM Corp. IBM SPSS Statistics for Windows, Version 22.0. Armonk, NY: IBM Corp (2013).

[21] Tessema DG Girma E Mekonnen TC Mebratu W. The extent of maternal nutritional knowledge and practice during lactation in Kombolcha town, south Wollo zone, Ethiopia: a mixed study design. Int J Women's Health. (2020) 12:79–87. doi: 10.2147/IJWH.S234398, 32161505 PMC7051894

[22] Bibi N Iftikhar K Fatima A Akmal A Akbar T Zafar M . Relationship between maternal nutritional knowledge and children's growth under 5 years of age in Lakhodair community, Lahore, Pakistan. Biol Clin Sci Res J. (2024) 2024:1121. doi: 10.54112/bcsrj.v2024i1.1121

[23] Debela BL Demmler KM Rischke R Qaim M. Maternal nutrition knowledge and child nutritional outcomes in urban Kenya. Appetite. (2017) 116:518–26. doi: 10.1016/j.appet.2017.05.042, 28558957

[24] Papežová K Kapounová Z Zelenková V Riad A. Nutritional health knowledge and literacy among pregnant women in the Czech Republic: analytical cross-sectional study. Int J Environ Res Public Health. (2023) 20:3931. doi: 10.3390/ijerph20053931, 36900942 PMC10001919

[25] Glick AA Winham DM Heer MM Hutchins AM Shelley MC. Nutrition knowledge varies by food group and nutrient among adults. Foods. (2025) 14:606. doi: 10.3390/foods14040606, 40002050 PMC11854791

[26] Sanlier N Kocaay F Kocabas S Ayyildiz P. The effect of sociodemographic and anthropometric variables on nutritional knowledge and nutrition literacy. Foods. (2024) 13:346. doi: 10.3390/foods13020346, 38275713 PMC10814858

[27] Marhamah M Emilia E Yusrafiddin Y Pakpahan SP Rassy RP. Relationship between employment status and knowledge of balanced nutrition among students with nutritional status. Sport Nutr J. (2023) 5:78–86. doi: 10.15294/spnj.v5i2.73484

[28] Esmat S Elhabashi E Sabry HA. Assessment of maternal nutritional knowledge and its predictors among mothers attending an urban primary health care unit in Giza. Egypt J Nutr Health. (2023) 17:35–50. doi: 10.21608/ejnh.2023.283071

[29] Teferi T Endalk G Ayenew GM Fentahun N. Inadequate dietary diversity practices and associated factors among postpartum mothers in Gambella town, Southwest Ethiopia. Sci Rep. (2023) 13:7252. doi: 10.1038/s41598-023-29962-6, 37142603 PMC10160103

[30] Ajzen I. The theory of planned behavior. Organ Behav Hum Decis Process. (1991) 50:179–211. doi: 10.1016/0749-5978(91)90020-T

[31] Demilew YM Alene GD Belachew T. Effect of guided counseling on dietary practices of pregnant women in west Gojjam zone, Ethiopia. PLoS One. (2020) 15:e0233429. doi: 10.1371/journal.pone.0233429, 32453774 PMC7250435

[32] Olajide BR Van Der Pligt P McKay FH. Cultural food practices and sources of nutrition information among pregnant and postpartum migrant women from low- and middle-income countries residing in high-income countries: a systematic review. PLoS One. (2024) 19:e0303185. doi: 10.1371/journal.pone.0303185, 38723007 PMC11081330

[33] Lamadah SM. Postpartum traditional beliefs and practices among women in Makkah Al Mukkaramah, KSA. Life Sci J. (2013) 10:838–47. Available online at: http://www.lifesciencesite.com

[34] Ramulondi M de Wet H Ntuli NR. Traditional food taboos and practices during pregnancy, postpartum recovery, and infant care of Zulu women in northern KwaZulu-Natal. J Ethnobiol Ethnomed. (2021) 17:15. doi: 10.1186/s13002-021-00451-2, 33743760 PMC7981893

[35] Li J Gray HL Kim S Park H Lee Y Lee H . Postpartum diet and the lifestyle of Korean and Chinese women: a comparative study. Front Public Health. (2022) 10:803503. doi: 10.3389/fpubh.2022.803503, 35462835 PMC9019053

[36] Jeong G Park SW Lee YK Ko SY Shin SM. Maternal food restrictions during breastfeeding. Korean J Pediatr. (2017) 60:70–6. doi: 10.3345/kjp.2017.60.3.70, 28392822 PMC5383635

[37] Newmom.me. Postpartum Traditions around the World - Part 1: India. (2023) Available online at: https://www.newmom.me/blog/postpartum-traditions-around-the-world-part-1-india (Accessed June 22, 2025).

[38] Tobing VY Afiyanti Y Rachmawati IN. Following the cultural norms as an effort to protect the mother and the baby during the perinatal period: an ethnographic study of women’s food choices. Enferm Clin. (2019) 29:831–6. doi: 10.1016/j.enfcli.2019.04.125

